# Identification of Sialyl-Lewis(x)-Interacting Protein on Human Spermatozoa

**DOI:** 10.3389/fcell.2021.700396

**Published:** 2021-07-20

**Authors:** Ying Wang, Weie Zhao, Si Mei, Panyu Chen, Tsz-Ying Leung, Cheuk-Lun Lee, William S. B. Yeung, Jian-Ping Ou, Xiaoyan Liang, Philip C. N. Chiu

**Affiliations:** ^1^Department of Obstetrics and Gynecology, Queen Mary Hospital, The University of Hong Kong, Hong Kong, Hong Kong; ^2^Department of Obstetrics and Gynecology, Center of Reproductive Medicine, Peking University Third Hospital, Beijing, China; ^3^The Sixth Affiliated Hospital of Sun Yat-sen University, Guangzhou, China; ^4^Department of Physiology, Medical College, Hunan University of Chinese Medicine, Changsha, China; ^5^Shenzhen Key Laboratory of Fertility Regulation, Department of Obstetrics and Gynecology, The University of Hong Kong-Shenzhen Hospital, Shenzhen, China; ^6^Center for Reproductive Medicine, The Third Affiliated Hospital, Sun Yat-sen University, Guangzhou, China

**Keywords:** zona pellucida, human spermatozoa, sialyl-Lewis(x), C1orf56, fertilization rate

## Abstract

Capacitated spermatozoa initiate fertilization by binding to the zona pellucida (ZP). Defective spermatozoa-ZP binding causes infertility. The sialyl-Lewis(x) (SLeX) sequence is the most abundant terminal sequence on the glycans of human ZP glycoproteins involving in spermatozoa-ZP binding. This study aimed to identify and characterize the SLeX-binding proteins on human spermatozoa. By using affinity chromatography followed by mass spectrometric analysis, chromosome 1 open reading frame 56 (C1orf56) was identified to be a SLeX-binding protein of capacitated spermatozoa. The acrosomal region of spermatozoa possessed C1orf56 immunoreactive signals with intensities that increased after capacitation indicating translocation of C1orf56 to the cell surface during capacitation. Treatment with antibody against C1orf56 inhibited spermatozoa-ZP binding and ZP-induced acrosome reaction. Purified C1orf56 from capacitated spermatozoa bound to human ZP. A pilot clinical study was conducted and found no association between the percentage of capacitated spermatozoa with C1orf56 expression and *in vitro* fertilization (IVF) rate in assisted reproduction treatment. However, the percentage of C1orf56 positive spermatozoa in the acrosome-reacted population was significantly (*P* < 0.05) lower in cycles with a fertilization rate < 60% when compared to those with a higher fertilization rate, suggesting that C1orf56 may have functions after ZP-binding and acrosome reaction. A larger clinical trial is needed to determine the possible use of sperm C1orf56 content for the prediction of fertilization potential of sperm samples.

## Introduction

Human oocytes are surrounded by a ∼7–20 μm thick porous cellular extracellular matrix termed zona pellucida (ZP), which is the main player in spermatozoa-oocyte interactions and species-specific fertilization. Spermatozoa-ZP binding is the first event in fertilization. Defective spermatozoa-ZP binding leads to infertility and is an important cause of reduced fertilization rates in assisted reproduction (Liu and Baker, 2000). A meta-analysis has shown high predictive power of spermatozoa-ZP binding on fertilization outcome (Oehninger et al., 2000). Defective spermatozoa-ZP binding is more frequent for men with abnormal semen parameters, especially those with severe teratozoospermia and oligozoospermia (Liu and Baker, 2004). Despite the importance of spermatozoa-ZP interaction, the mechanisms regulating the process are unclear partly due to failure in the identification of ZP receptor(s) on human spermatozoa.

Human ZP is composed of four glycoproteins namely ZP glycoprotein 1 (ZP1), ZP2, ZP3, and ZP4 (Chiu et al., 2014). Glycan chains are found on the asparagine (N-linked) and serine/threonine (O-linked) residues of these ZP glycoproteins. We have identified sialyl-Lewis(x) (SLeX) [NeuAcα2–3Galβ1-4(Fucα1–3)GlcNAc] as the most abundant terminal sequence on the N-linked glycans of human ZP (Pang et al., 2011). Human oocyte–spermatozoon binding involves both protein-protein and protein-glycans interactions (Chiu et al., 2014; Gupta, 2021).

Recent transgenic mice studies suggested that ZP2 is the primary ligand for human sperm binding to ZP (Baibakov et al., 2012; Avella et al., 2014, 2016). Human spermatozoa bind to and penetrate the ZP of oocytes from transgenic mice carrying the four human ZP glycoproteins in place of the three mouse ZP glycoproteins (Baibakov et al., 2012). When human ZP2 is absent from these mice, human spermatozoa rarely bind to the oocytes (Avella et al., 2014). It is noted that the human spermatozoa take 180–240 min to bind onto the mouse ZP containing human ZP2 (Baibakov et al., 2012), which was much longer than 15–60 min for binding to human ZP (Plachot et al., 1986; Morales et al., 1994). The slower binding kinetics could be due to inappropriate glycosylation of human ZP2 in the mice, consistent with differential interaction of human spermatozoa to recombinant human ZP2 and native ZP2 with different glycosylation (Chiu et al., 2014; Gupta, 2021).

Several glycoconjugates have been associated with human spermatozoa-ZP binding (Chiu et al., 2014; Gupta, 2021). For example, glycoconjugates terminated with the SLeX sequences or antibodies against the sequence inhibited the spermatozoa-ZP binding (Pang et al., 2011). Synthesized highly complex triantennary N-glycans with SLeX moieties have increased inhibitory activities (Chinoy and Friscourt, 2018). Spermatozoa-SLeX interaction has been used to capture human spermatozoa in a microfluidic system for forensic investigation (Inci et al., 2018; Deshmukh et al., 2020). The ZP glycans may take part in direct interaction with the sperm ZP receptors or may provide the proper tertiary structure that maximizes the availability of the ZP glycoproteins to their receptors on spermatozoa.

The identity of human sperm ZP receptor(s) is controversial (Gupta, 2018; Tumova and Zigo, 2021). Several human sperm carbohydrate-binding proteins have been proposed (Chiu et al., 2007b). However, antibodies against and competitors/substrates of these molecules fail to completely block spermatozoa-ZP binding and/or ZP-induced acrosome reaction, suggesting that they are not solely mediating spermatozoa-ZP binding. The objective of this study was to identify and characterize the human sperm SLeX-binding proteins.

## Materials and Methods

### Semen and Oocyte Samples

The Ethics Committee of the University of Hong Kong/Hospital Authority Hong Kong West Cluster approved the research protocol. Informed consent was obtained from patients who donated their semen and oocyte samples for research use. Human spermatozoa were collected by masturbation from patients attending the infertility clinic at the Queen Mary Hospital and the Family Planning Association, Hong Kong (Mei et al., 2019). Only semen samples with normal semen parameters according to the World Health Organization (2010) criteria (strict criteria >4%, volume >1.5 ml, total sperm number >39 × 10^6^ per ejaculate, concentration >15 × 10^6^/ml, total motility >40%, progressive motility >32%, and vitality >58%) were used. Spermatozoa were processed by density gradient centrifugation using AllGrad (LifeGlobal, Brussels, Belgium) and resuspended in Earle Balanced Salt Solution (EBSS; Flow Laboratories, Irvine, United Kingdom) supplemented with 0.3% bovine serum albumin (BSA), 0.3 mmol/l sodium pyruvate, 0.16 mmol/l penicillin-G, 0.05 mmol/l streptomycin sulfate, and 14 mmol/l sodium bicarbonate (all from Sigma, St Louis, MO, United States) (EBSS/0.3% BSA) to a concentration of 2 × 10^6^ spermatozoa/ml. Capacitated spermatozoa were prepared by a 3-h incubation in EBSS supplemented with 3% BSA as previously described (Chiu et al., 2010).

For the pilot clinical study, semen samples were collected from men whose female partner underwent conventional IVF treatment at the Sixth affiliated Hospital of the Sun Yat-sen University, Guangzhou, China. The research protocol of the study was approved by the Ethics Committee of the Sun Yat-sen University. Semen samples were prepared by density gradient centrifugation followed by a standard swim-up procedure World Health Organization (2010). Briefly, 1 mL EBSS/0.3%BSA medium was gently layered over the processed spermatozoa pellet in a sterile 15 mL centrifuge tube after density gradient centrifugation. The tube was inclined at 45° and incubated for 1 h at 37°C. The upper 0.5 mL medium was collected for IVF treatment and the remaining sperm suspension was used for experimentation. After washing with EBSS/0.3%BSA once, the final sperm pellet was resuspended in 1 mL EBSS/0.3%BSA. The motility and morphology of the spermatozoa were then determined.

Human oocytes were collected from the Assisted Reproduction Program at the Queen Mary Hospital, Hong Kong. Unfertilized metaphase II oocytes from couples who underwent intracytoplasmic sperm injection were collected and stored in an oocyte storage buffer at 4°C. The oocyte storage buffer contained 1.5 M MgCl_2_, 0.1% polyvinyl pyrrolidone (PVP) and 40 mM HEPES with pH 7.2.

### Determination of Sperm Viability

Sperm viability was determined by the trypan blue exclusion staining. Processed spermatozoa (2 × 10^5^) and trypan blue dye were mixed in a 1:1 ratio to a final volume of 12 μL. The mixture was placed on a glass slide for 3 min before examination under a light microscope with 400 × magnification. Viable spermatozoa were transparent without staining, whereas non-viable spermatozoa were stained blue. At least 200 spermatozoa were randomly counted to evaluate the sperm viability of the sample.

### Determination of Sperm Motility

The Hobson sperm tracker system (Hobson Tracking Systems Ltd., Sheffield, United Kingdom) was used to determine the sperm motility parameters in all the experiments except the clinical study. The set-up parameters of the system and the procedures were described previously (Huang et al., 2013). Each measurement was performed on a warmed microscope stage at 37°C. Five hundred spermatozoa per sample in randomly selected fields were evaluated to determine (1) average path velocity (VAP, μm/s), (2) curvilinear velocity (VCL, μm/s), (3) straight-line velocity (VSL, μm/s), (4) beat cross frequency (BCF, Hz), (5) amplitude of lateral head displacement (ALH, μm), (6) mean linearity (LIN, VSL/VCL), (7) straightness (STR, VSL/VAP), (8) percentage hyperactivation (HYP), and (9) percentage progressive motility (VAP ≥ 25 μm/s). All samples were processed in triplicate.

Manual sperm motility counting based on the WHO recommended protocol (World Health Organization, 2010) was also used. In manual counting, 2 × 10^5^ processed spermatozoa were added on a glass slide and observed under a light microscope at 400 × magnification. At least 200 spermatozoa were counted randomly. Their motilities were divided into three classes according to the WHO criteria (World Health Organization, 2010), namely progressive motile (PR), non-progressive motile (NP) and immotile (IM). PR were spermatozoa moving actively, either linearly or in a large circle. NP were those exhibiting all other patterns of motility and without progression; IM were those without movement. In this study, total motility referred to the sum of PR and NP spermatozoa. The WHO reference value of total motility is >40%. For comparison purposes, the samples were divided into a high motility group and a low motility group when their total motility was >40% and ≤40%, respectively.

### Determination of Sperm Morphology

Diff-Quik staining was used to determine sperm morphology. In general, 10 μL of semen was smeared on a glass slide, air-dried at room temperature and placed successively in the Diff-Quik fixative (Microptic S.L., Barcelona, Spain) for 10 s, the Diff-Quik solution I for 15 s, and the Diff-Quik solution II for 15 s at room temperature. The slides were then placed under running water to remove excess stain and air-dried at room temperature. The morphology of spermatozoa was determined under a light microscope at 1,000 × magnification. For each sample, 200 spermatozoa in randomly selected fields were counted. Only spermatozoa with both head and tail seen were assessed. The samples were considered normal when the percentage of spermatozoa with normal morphology as defined by strict criteria (World Health Organization, 2010) was >4%.

### Hemizona Binding Assay

The hemizona binding assay was performed as described (Yao et al., 1998). Unfertilized oocytes were micro-bisected into two identical hemizona. Each hemizonae was incubated with 2 × 10^5^ spermatozoa/mL in a 100 μL droplet of EBSS/0.3%BSA under mineral oil for 3 h at 37°C in an atmosphere of 5% CO_2_. After incubation, the loosely bound spermatozoa were removed by several washes with EBSS/0.3% BSA and the numbers of tightly bound spermatozoa on the outer surface of the hemizona were counted. The results are expressed as hemizona index (HZI), which is the ratio of the number of bound spermatozoa in the test droplet to that in the control droplet × 100.

### Determination of Ionophore and ZP-Induced Acrosome Reaction

Purification of solubilized ZP was performed as described (Chiu et al., 2010). Briefly, the ZP was separated from the oocytes under a microscope and heat-solubilized at 70°C in 5 mM NaH_2_PO_4_ buffer (pH 2.5) for 90 min. Capacitated spermatozoa at a concentration of 2 × 10^6^ spermatozoa/ml were incubated with solubilized ZP (1 μg/ml, 60 min) or ionophore A23187 (2.5 μM, 30 min) at 37°C in an atmosphere of 5% CO_2_ in air (Chiu et al., 2008a). The acrosomal status of the treated spermatozoa was then evaluated.

### Determination of Acrosomal Status

The percentage of capacitated spermatozoa was assayed by chlortetracycline staining (CTC) as described (Chiu et al., 2005). The capacitation status of 200 spermatozoa were evaluated under a fluorescence microscope (Zeiss, Oberkochen, Germany) at × 630 magnification. Five CTC staining patterns of the sperm head were identified (Chiu et al., 2005). CTC4 pattern (uniform head fluorescence) was the main capacitated pattern.

Fluorescein isothiocyanate-labeled *Pisum sativum* (FITC-PSA) (Sigma, St Louis, MO, United States) was used to determine acrosome reaction. Processed spermatozoa (0.5 × 10^6^) were fixed in 300 μl of 95% ethanol and dried on slides before staining with Hoechst 33258 [Pentahydrate (bis-Benzimide); Sigma] and 2 μg/20 μL FITC-PSA in PBS for 30 min. The slides were then washed, mounted in glycerol containing 0.2 M n-propyl gallate (Sigma), and observed under a fluorescent microscope (Zeiss). At least 200 spermatozoa were randomly selected and counted under the microscope with 400 × magnification. Acrosome-reacted spermatozoa were defined as those without Hoechst and FITC-PSA staining or with FITC-PSA staining at the equatorial segment only. The filter set used for CTC and FITC-PSA staining consisted of an excitation filter BP 450-490, a chromatic beam splitter FT510 and a barrier filter LP520.

### Purification of Sialyl-Lewis(x) Binding Protein

Sialyl-Lewis(x) -binding proteins on the plasma membrane of capacitated spermatozoa were identified by our established chromatographic method using SLeX-BSA neoglycoprotein affinity column followed by mass spectrometric analysis as described (Chiu et al., 2007a; Lee et al., 2011). In brief, capacitated spermatozoa (100 × 10^6^) were washed thrice in EBSS. Non-integral, peripheral membrane-associated proteins on spermatozoa were removed by incubation of the washed spermatozoa in 1 M NaCl in PBS with gentle stirring for 10 min at 25°C as described (Chiu et al., 2007a). The spermatozoa were then collected by centrifugation at 600 × *g* for 10 min before extraction of the sperm plasma membrane proteins by the ProteoExtract Native Membrane Protein Extraction Kit (Merck, Kenilworth, NJ, United States) according to the manufacturer’s instructions. The insoluble fraction was discarded after centrifugation at 15,000 × *g* for 40 min. The supernatant was diluted in a solution of MOPS-NaOH buffer (pH 7.3) containing 0.2% Triton X-100 and 6 mM MnCl_2_. SLeX-BSA (Dextra, Reading, United Kingdom) conjugated sepharose beads (GE Healthcare) were used to precipitate the SLeX-binding protein from the extracted membrane protein fractions. Lewis(x) (LeX)-BSA, which did not bind to human spermatozoa (Chiu et al., 2012) was used as a control.

### Tryptic In-Solution Digestion

The purified SLeX-binding proteins were precipitated in precooled acetone and collected as a pellet after centrifugation at 16,000 × *g* for 10 min followed by removal of the supernatant. Trypsin digestion of the pellets was performed as described (Ye et al., 2015). The digestion was terminated by acidification with 1% trifluoroacetic acid. The supernatant was then saved until use.

### Two-Dimensional Liquid Chromatography With Tandem Mass Spectrometry

The Proteomic Laboratory for System Biology Research (Baptist University, Hong Kong, People’s Republic of China) performed 2D LC-MS/MS analyses on the purified samples. The liquid chromatographic separation was conducted in a nano-liquid chromatography system (Dionex UltiMate 3000 Nano-LC System; Dionex). For the first dimension, two buffers were used to set up a gradient for separation: buffer A was 20% acetonitrile/10 mM potassium dihydrogen phosphate, pH 3.0; buffer B was buffer A plus 0.5 M potassium chloride. Peptides were injected into a 50 × 1.0 mm polysulfoethyl A strong cation exchange column (Poly LC Inc.) and eluted at a flow rate of 30 μL/min with increasing salt concentrations (100% buffer A and 0% buffer B from 0 to 10 min; 70% buffer A and 30% buffer B from 10 to 40 min; 65% buffer A and 35% buffer B from 40 to 45 min, 0% buffer A, and 100% buffer B from 45 to 50 min). Fractions were collected every 5 min.

The 2D LC separation was performed in the same nano-LC system with a reverse-phase column (Pepmap C18, 75 μm × 150 mm; Dionex). Two buffers were used for this step: buffer C was 0.05% trifluoroacetic acid and buffer D was 80% ACN/0.04% trifluoroacetic acid. Each fraction from the strong cation exchange column was injected into a reversed-phase column with a 100-min linear gradient (a 90-min gradient from 0 to 35% buffer D; 5 min from 35% buffer D to 50% buffer D; and a 5-min holding at 90% buffer D). Matrix-assisted laser desorption ionization (MALDI) spot was applied every 30 s, and the MS/MS analysis was performed in a Bruker Autoflex III MALDI Tandem Time-of-Flight (MALDI TOF/TOF) Mass Spectrometer (Bruker Daltonics).

### Analysis of MS Data

Mascot (version 2.2.04^1^) was used to identify the peptides. Each MS/MS spectrum was searched against the Human IPI Protein Database 3.71. Proteins were considered to be successfully identified when the total Mascot score reached 65 or above.

### Expression and Localization of Sperm C1orf56

Uncapacitated and capacitated spermatozoa (2 × 10^6^) were extracted by SDS, resolved by 10% SDS-PAGE and blotted on polyvinylidene difluoride membranes. Western blotting was performed using a rabbit polyclonal anti-chromosome 1 open reading frame 56 (C1orf56) antibody (0.1 μg/mL; Sigma). Anti-beta-tubulin antibody (Sigma) was used to determine sample loading. Quantification of protein bands normalized with respect to the tubulin control was carried out with the Image J 1.49 software^2^.

For immunostaining and cytometry analysis, human spermatozoa were mildly fixed in 0.5% paraformaldehyde for 10 min at room temperature (Chiu et al., 2008b) followed by incubation with polyclonal anti-C1orf56 antibody (1 μg/mL; Sigma) overnight at 4°C. The bound antibodies were detected by the Alexa Fluor 488/555-conjugated goat anti-rabbit IgG (Invitrogen, CA, United States). For simultaneous staining, the slides were washed, further immersed for 1 min in ice-cold methanol for cell permeabilization and incubated with FITC-PSA for 9 min following our established protocol (Chiu et al., 2008b). After washing, the spermatozoa were examined under a fluorescence microscope (Zeiss) with 600 × magnification or a flow cytometer (BD FACSCanto II Analyzer; BD Biosciences). The flow cytometry data were evaluated with the use of the Flowjo software (Tree Star).

### Effects of Anti-C1orf56 Antibody on Sperm Functions

Capacitated spermatozoa (2 × 10^6^/mL) were pre-incubated in medium supplemented with functional blocking anti-C1orf56 antibody (0.01, 0.1 or 1 μg/mL; Sigma) at 37°C in a 5% CO_2_ atmosphere for 1 h. Isotypic-matched antibody (non-specific rabbit IgG; Invitrogen) was used as control. The spermatozoa were washed with fresh EBSS/0.3% BSA before evaluation of their viability and motility, acrosomal status, ZP-induced acrosome reaction and ZP binding capacity as described above. To study the effect of anti-C1orf56 antibody treatment on the binding capabilities of the capacitated spermatozoa to SLeX, capacitated spermatozoa (5 × 10^7^) were incubated with 0.5 μM Alexa Fluor-594 labeled SLeX-BSA in the presence of 1 μg/ml anti-C1orf56 antibody for 120 min followed by the flow cytometry analysis.

### Purification of C1orf56 on Human Spermatozoa

C1orf56 on human spermatozoa was purified by immuno-affinity chromatography. To prepare the anti-C1orf56 affinity column, cyanogen bromide activated Sepharose 4 Fast Flow gel beads (GE Healthcare) were swelled in the swelling buffer (1 mM HCl) for 30 min, and washed with 10 gel volumes of the same buffer. Antibody coupling was done by incubation of 1 mg of anti-C1orf56 antibodies (Sigma) with 0.5 mL of swelled cyanogen bromide beads in coupling buffer overnight at 4°C with gentle shaking. The coupled affinity column was then washed with blocking buffer (0.2 M glycine, pH 8.0) before further blocking in the same buffer overnight at 4°C. The blocked affinity column was then washed successively with the coupling buffer and the acetate buffer (0.1 M sodium acetate, 0.5 M NaCl, and pH 4.0) for four times. Finally, the affinity column was washed twice with Tris-buffered saline (TBS: 25 mM Tris, 3 mM KCl, and 140 mM NaCl, pH 7.4; USB corporation, Cleveland, OH, United States) and kept at 4°C until use.

The extracted membrane proteins (see above) of capacitated spermatozoa (5 × 10^7^) were loaded onto the anti-C1orf56 antibody coated Sepharose column and washed successively by TBS, 1 M NaCl with 1% isopropyl alcohol, 10 mM ammonium acetate with 0.1% isopropyl alcohol (pH 5.0), and TBS. The bound C1orf56 was eluted with 0.1% trifluoroacetic acid. The concentration of the purified C1orf56 was determined by a protein assay kit (Bio-Rad, Hercules, CA, United States). The purity of C1orf56 was checked by SDS-PAGE and western blotting.

### Binding of C1orf56 to Zona Pellucida

Purified C1orf56 was labeled with Alexa Fluor-594^®^ (Alexa Fluor 594 Protein Labeling Kit; Molecular Probes). Matched hemizona were incubated with 1 μg/mL labeled C1orf56 in the presence or absence of the anti-C1orf56 neutralizing antibody (Sigma) for 3 h at 37°C in an atmosphere of 5% CO_2_. The binding was then observed under a fluorescence microscope.

### Ovarian Stimulation and *in vitro* Fertilization

Couples attending the infertility clinic at the Sixth Affiliated Hospital of Sun Yat-sen University from January, 2016 to March, 2017 were recruited in this study. The standard gonadotrophin-releasing hormone agonist long protocol was used. Conventional insemination was performed 4 h after oocyte retrieval, and the fertilization check was conducted after 16–18 h. Normal fertilization was indicated by the appearance of two pronuclei (2PN). Fertilization rate was defined as the number of 2PN zygotes observed divided by the total number of inseminated oocytes × 100.

### Data Analyses

All values were expressed as mean ± standard error of the mean (SEM). For all experiments, the non-parametric rank sum test for comparisons was used to identify differences between groups. If the data were normally distributed, parametric Student *t*-test was used as the posttest. The data were analyzed by SPSS 20.0 (IBM) and *P*-value < 0.05 was considered as statistically significant.

## Results

### Identification of Potential SLeX-Binding Proteins

**FIGURE 1:**
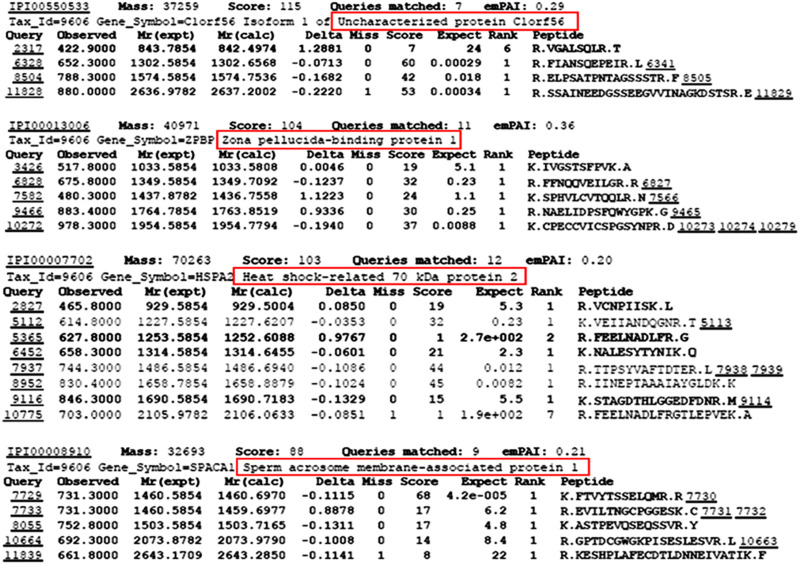
Identification of SLeX/LeX-binding proteins from sperm membrane protein extracts using SLeX/LeX-BSA affinity chromatography followed by MALDI-TOF-MS/MS. Sperm membrane protein extracts were purified by membrane protein extraction kit (ProteoExtract native membrane protein Extraction Kit; Merck). Each MS/MS spectrum was searched against the Human IPI Protein Database 3.71.

Mass spectrometry analysis of the SLeX-BSA affinity purified sperm membrane fraction identified a total of 59 proteins with Mascot protein score higher than 65^3^. Proteins were selected for studies when they were absent in the LeX-BSA (Dextra) affinity purified fraction and when they had been reported to be present only in human sperm head region. Only 4 proteins met these criteria. They were chromosome 1 open reading frame 56 (C1orf56), ZP-binding protein 1 (ZPBP1), heat shock-related 70 kDa protein 2 (HSPA2) and sperm acrosome membrane-associated protein 1 (SPACA 1) (Figure 1). ZPBP1 (Lin et al., 2007), HSPA2 (Huszar et al., 2000), and SPACA1 (Hao et al., 2002) are known to be involved in spermatozoon-oocyte interaction. In this report, C1orf56 was investigated for its role in spermatozoa-ZP interaction.

### Presence of C1orf56 in Human Spermatozoa

**FIGURE 2:**
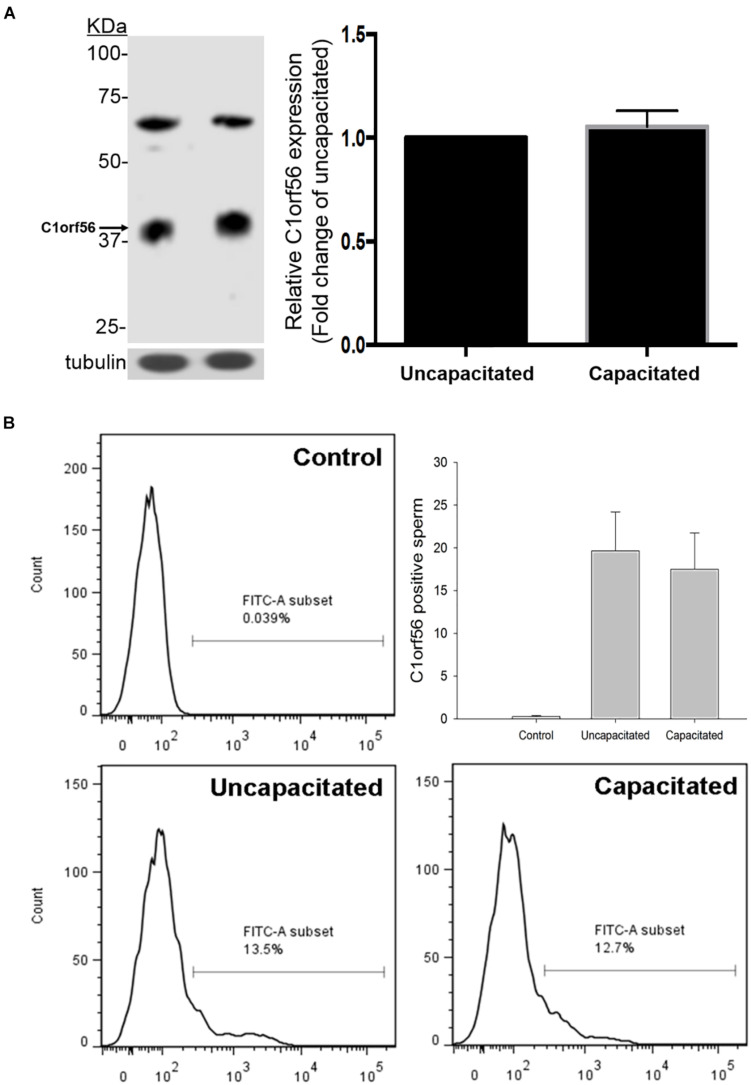
Uncapacitated and capacitated spermatozoa possess similar levels of C1orf56 expression. **(A)** Washed spermatozoa (2 × 10^6^) were lysed and resolved in 10% SDS-PAGE followed by Western blotting using anti-C1orf56 antibody. Sample loading was revealed by anti-tubulin antibody. Semi-quantitative comparison of C1orf56 expression between uncapacitated and capacitated sperm samples in Western blotting was also shown. **(B)** Flow cytometry analysis of the C1orf56 surface expression on uncapacitated and capacitated sperm. Spermatozoa were first incubated with 1 μg/mL anti-C1orf56 antibody or isotypic-matched antibody from the same species (control) followed by Alexa Fluor-488 fluorescence-conjugated secondary antibody. All data are represented as mean ± standard error of the mean (SEM) (*N* = 5).

The percentage of capacitated spermatozoa increased from 16.86 ± 3.0% to 61.43 ± 4.8% (*N* = 10) (Supplementary Figure 1) after capacitation, as demonstrated by chlortetracycline staining (Chiu et al., 2005). The anti-C1orf56 antibody recognized a major protein band of size ∼39 kDa in the human sperm extract (Figure 2). Densitometric analysis of Western blot (Figure 2A) showed the presence of a comparable amount of C1orf56 in the uncapacitated and capacitated spermatozoa. Another band of 65 kDa was also found which might represent the non-specific binding of the antibodies to the albumin which were abundant in the sperm culture medium. Similar observation on the sperm C1orf56 expression was found in flow cytometric analysis (Figure 2B); the percentage of uncapacitated and capacitated spermatozoa with positive C1orf56 immunoreactivities were 19.6 ± 4.6% and 17.5 ± 4.2%, respectively.

**FIGURE 3:**
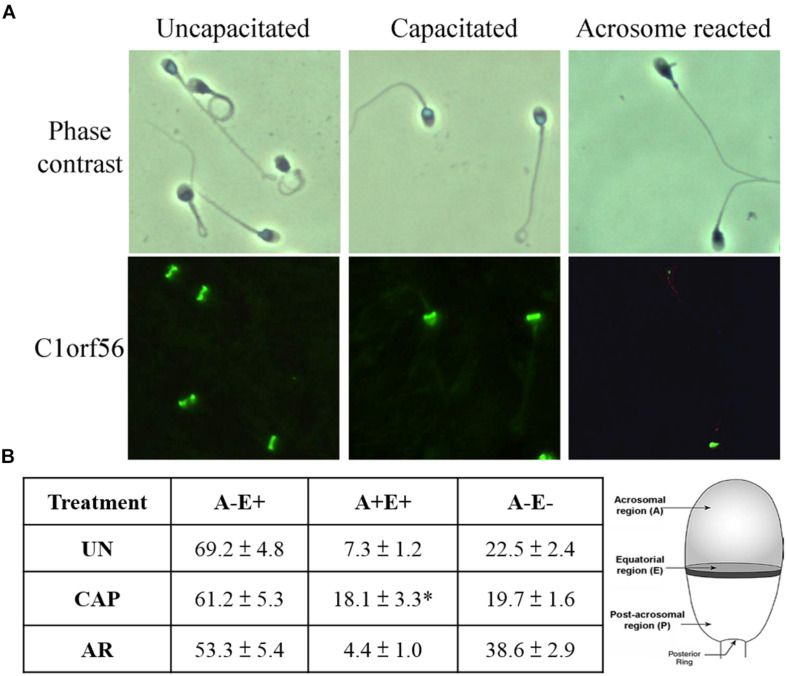
Acrosomal region of human spermatozoa possesses strong C1orf56 immunoreactivity after capacitation. **(A)** C1orf56 immunoreactivities were visualized using Alexa Fluor 488-conjugated secondary antibody. Three staining patterns were observed in the C1orf56-positive sperm: (A–E+): Focal signals over the equatorial region; (A+E+): Strong signals over both the equatorial and acrosomal regions; (A–E–): No staining signal on sperm head. All the results shown are representative of five replicate experiments. **(B)** Percentage of C1orf56-positive sperm with A–E+, A+E+, or A–E– staining patterns. 200 C1orf56-positive sperm in randomly selected fields were determined under a fluorescence microscope after immunostaining. All data are represented as mean ± standard error of the mean (SEM). *P < 0.05 when compared the percentages of A+E+ staining pattern of C1orf56 in capacitated (CAP) sperm with uncapacitated (UN) or acrosome reacted (AR) sperm. A, acrosomal region; E, equatorial region, *N* = 10.

Immunostaining for C1orf56 was performed on non-permeabilized uncapacitated, capacitated and acrosome reacted spermatozoa (Figure 3). Most uncapacitated C1orf56-positive spermatozoa (69.2 ± 4.8%) exhibited immunoreactive signals on the equatorial region. After capacitation, strong signals were observed in the acrosomal region (18.1 ± 3.3%). Calcium ionophore treatment significantly induced acrosome reaction of the capacitated spermatozoa (7.62 ± 1.9% vs. 45.82 ± 7.8%; *N* = 5) (Supplementary Figure 1). The fluorescence signals on the acrosomal region became much weaker after ionophore-induced acrosome reaction (Figure 3B). This observation was further confirmed by simultaneous staining with antibody against C1orf56 and FITC-PSA (Supplementary Figure 2). The observation suggests translocation of C1orf56 to sperm surface during capacitation, which is lost after acrosome reaction.

### Anti-C1orf56 Antibody inhibited Spermatozoa-Zona Pellucida and -SLeX Binding

**FIGURE 4:**
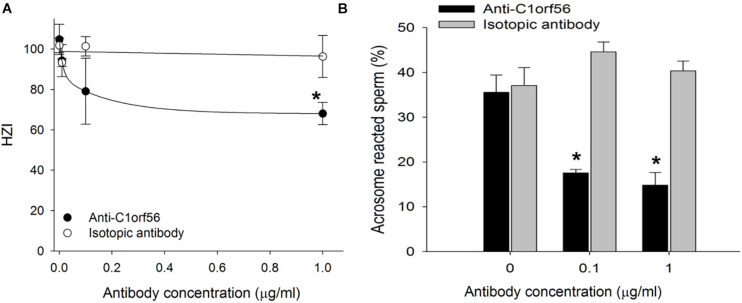
Anti-C1orf56 antibody inhibits spermatozoa-ZP interaction. Capacitated spermatozoa were incubated in culture medium supplemented with different concentrations of anti-C1orf56 antibody. The **(A)** ZP-binding capacity and **(B)** solubilized ZP-induced acrosome reaction of the sperm were then determined by hemizona assay and FITC-PSA staining, respectively. Hemizona binding index (HZI) was the ratio of the number of bound spermatozoa on the test hemizona to that on the control hemizona times 100. *P < 0.05 when compared with the corresponding control with isotypic matched antibody.

**FIGURE 5:**
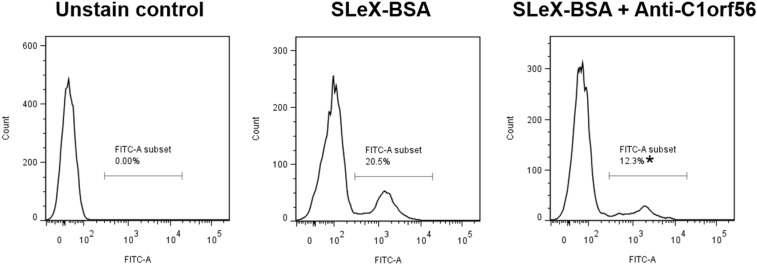
Anti-C1orf56 antibody inhibits spermatozoa-SLeX binding. Capacitated sperm were incubated with 0.5 μM Alexa Fluor-594 labeled SLeX-BSA in the presence or absence of 1 μg/ml anti-C1orf56 antibody for 120 min followed by the flow cytometry analysis. The results shown are representative of three replicate experiments. *P < 0.05 when compared with the control without anti-C1orf56 antibody.

Treatment with anti-C1orf56 antibody at a concentration of 1 μg/ml significantly (*P* < 0.05) decreased the number of capacitated spermatozoa bound onto hemizona (Figure 4A) when compared to the untreated control spermatozoa or those treated with isotypic-matched antibody. The treatment also suppressed spermatozoa-SLeX binding (Figure 5) and ZP-induced acrosome reaction of capacitated spermatozoa (Figure 4B) significantly. The antibody at the concentration used did not affect sperm viability, motility and acrosomal status (Supplementary Figures 3–5).

### Purified C1orf56 Bound to Human Zona Pellucida

**FIGURE 6:**
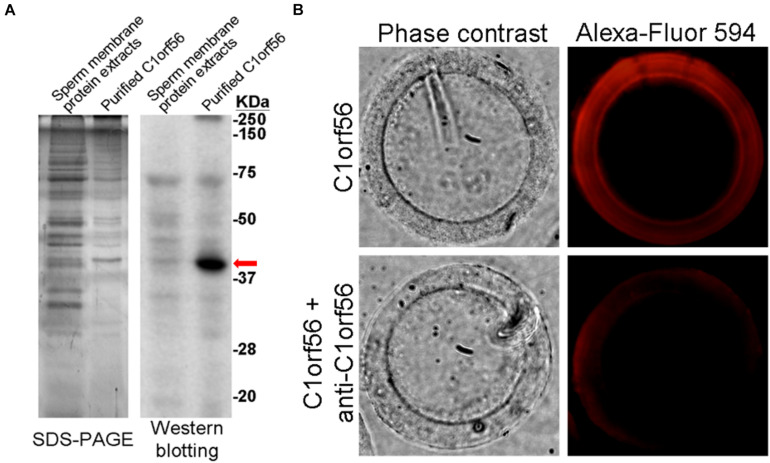
Binding of purified C1orf56 to human ZP. **(A)** C1orf56 was partially purified from membrane protein extracts of capacitated spermatozoa by anti-C1orf56 immuno-affinity chromatography. The purity of the purified proteins (arrow) was checked by SDS-PAGE and Western blotting. **(B)** Purified C1orf56 was labeled with Alexa Fluor-594 labeling kit (Invitrogen). Matching hemizona were incubated with the 1 μg/mL labeled C1orf56 in the presence or absence of polyclonal anti-C1orf56 neutralizing antibody. The results shown are representative of three replicate experiments.

C1orf56 with molecular size ∼39 kDa was significantly enriched from spermatozoa by affinity chromatography (Figure 6A). Fluorescence-labeled C1orf56 bound to the ZP of human oocytes specifically (Figure 6B). There were impurities in the partially purified C1orf56 fraction as demonstrated by SDS-PAGE. The impurities may be due to non-specific interaction of the cyanogen bromide activated Sepharose 4 with other proteins eluted together with C1orf56 during purification (Kennedy and Barnes, 1980). In order to demonstrate the specific action of c1orf56 on binding to ZP, we included a control study using neutralizing antibody against c1orf56 (Figure 6B). The bound signal diminished in the presence of the neutralizing antibody. To increase the purity of the isolated C1orf56, further purification steps, such as ion exchange chromatography, and gel filtration, were required.

### The Relationship Between C1orf56 Surface Expression and Fertilization Rates

**FIGURE 7:**
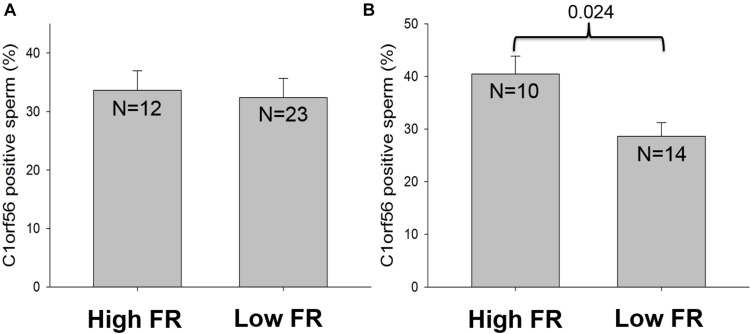
The relationship between C1orf56 expression and fertilization rate. C1orf56 surface expression is determined on **(A)** capacitated spermatozoa and **(B**) acrosome reacted spermatozoa by flow cytometry. Data was classified and analyzed according to the fertilization rate (FR), which is divided into high FR group (*FR* ≥ 60%) and low FR group (*FR* < 60%).

The association of C1orf56 surface expression with fertilization rate (FR) was investigated. Flow cytometry analysis was used to detect the surface expression of C1orf56 in spermatozoa. In this analysis, the samples were divided into a high FR group with fertilization rate is ≥60% and a low FR group with fertilization rate <60%. There was no significant difference in the percentage of capacitated spermatozoa with surface C1orf56 expression between the high and the low FR group (*P* > 0.05, Figure 7A). In the acrosome reacted spermatozoa population (Figure 7B), there was a significantly lower C1orf56 expression in the low FR group when compared to the high FR group.

The relationship between the C1orf56 expression and sperm motility and morphology were also studied. When the samples were divided into a high motility group (Total motility >40%) and a low motility group (total motility ≤40%), there was no difference between the two groups (Supplementary Figure 6). Similarly, there was no difference in both the capacitated and the acrosome reacted subpopulation between samples with percentages of normal form >4% and ≤4% (Supplementary Figure 7).

## Discussion

Using an affinity chromatography with SLeX as a bait, four potential SLeX-binding proteins, including chromosome 1 open reading frame 56 (C1orf56), was identified to be the SLeX-binding proteins of capacitated spermatozoa. The contribution of C1orf56 to spermatozoa-ZP interaction was further demonstrated by the binding of purified C1orf56 to the ZP as well as the inhibitory effect of anti-C1orf56 antibody on spermatozoa-ZP interaction.

This is the first study on localization and function of C1orf56 in human spermatozoa. C1orf56 was identified in a human sperm proteomic study (Wang et al., 2013). Immunohistochemical staining of human testicular tissue shows strong C1orf56 immunoreactivities only in cells of the seminiferous tubules (The Human Protein Altas^4^), suggesting a role of C1orf56 in sperm functions. Consistently, we demonstrated that human sperm surface C1orf56 is involved in spermatozoa-ZP binding.

Derived from three observations: (1) Immunofluorescence staining localized C1orf56 to the acrosomal region of capacitated spermatozoa, a region that binds ZP (Chiu et al., 2008b); (2) Anti-C1orf56 antibody suppressed spermatozoa-SLeX and spermatozoa-ZP binding and ZP-induced acrosome reaction; and (3) Purified C1orf56 from spermatozoa bound to human ZP. The mechanism by which C1orf56 regulates spermatozoa-SLeX binding is unknown. Bioinformatics analysis using a motif scan program revealed that C1orf56 contains a thrombospondin type-1 (TSP1) repeat profile known to be involved in the binding of multiple matrix glycoproteins and proteoglycans (Adams and Tucker, 2000). The possible involvement of TSP1 repeats on ZP binding needs further investigation.

According to the contact mechanics theory (Kozlovsky and Gefen, 2012), a high density of sperm ZP receptors on the sperm head is required to provide sufficient biochemical binding forces for efficient spermatozoa-ZP interaction counteracting the propulsive forces generated by the swimming spermatozoa. Despite the theoretical need of a large number of ZP receptors on spermatozoa for fertilization, their identities are controversial. Several candidate carbohydrate-binding proteins such as fucosyltransferase-5 (Chiu et al., 2007b), sperm agglutination antigen-1 (Diekman et al., 1997), alpha-D-mannosidase (Tulsiani et al., 1990), and galactosyltransferase (Shur et al., 1998), have been proposed as the putative receptors on human spermatozoa. The failure of genetic ablation of these potential molecules in affecting male fertility in animal models, and the inability of antibodies against and competitors/substrates of these molecules to completely block human spermatozoa-ZP binding and/or ZP-induced acrosome reaction, suggest that they are not the sole mediator of spermatozoa-ZP binding and that there are multiple sperm receptors for the ZP glycoproteins (Wassarman, 1999; Wassarman et al., 2001; Avella et al., 2013). Consistently, anti-C1orf56 antibody alone could not completely block spermatozoa-ZP binding.

Apart from C1orf56, three other SLeX-binding proteins were identified in this study. ZPBP1 (Lin et al., 2007) and SPACA1 (Hao et al., 2002) are involved in spermatozoon-oocyte interaction in animals, but there are no similar studies on human spermatozoa. HSPA2 is a testis-enriched member of the heat shock protein family. In humans, HSPA2 facilitates the assembly and/or presentation of ZP-interacting protein complexes on the sperm surface (Nixon et al., 2015). Interestingly, the HSPA2-associated ZP-interacting complex undergoes a capacitation-associated translocation to the outer leaflet of the sperm surface (Nixon et al., 2015). Reduced expression of HSPA2 from the human sperm proteome reduces the capacity for spermatozoa-oocyte recognition and fertilization after assisted reproduction treatment (Huszar et al., 2000). These data support a multi-molecular structures of the sperm ZP receptor(s) that are assembled during capacitation.

A human sperm ZP receptor complex has been identified. It composes of arylsulfatase A (ARSA), sperm adhesion molecule 1 (SPAM1) and HSPA2 (Redgrove et al., 2013). During capacitation, the complex is translocated to the sperm acrosomal region. ARSA mediates sperm-ZP interaction, SPAM1 is involved in the dispersal of cumulus matrix, and while HSPA2 organizes other proteins in the complex to be on the sperm surface (Asquith et al., 2004; Redgrove et al., 2012, 2013). Another sperm ZP receptor complex composing of galactosyltransferase (GalT) and SED1 has been reported (Shur et al., 2006). In this complex, GalT recognizes the ZP glycans while SED1 mediates the initial docking of the spermatozoa with the ZP to facilitate the GalT-ZP interaction. The relationship between C1orf56 and other sperm-ZP interacting proteins remains to be investigated.

The present results showed that C1orf56 expression is relocated from the equatorial region to the acrosomal region after capacitation. Capacitation involves lipid remodeling with rearrangement of glycoproteins on the sperm plasma membrane (Gadella et al., 2008; Fraser, 2010). The mechanism for the protein relocation is unknown. The lipids in the plasma membranes are organized as compact structures with microdomains termed lipid rafts, which are small, heterogeneous, highly dynamic, sterol and sphingolipid-enriched membrane domains formed through protein, and lipid interaction for cell adhesion and signaling (Harris and Siu, 2002; Lajoie et al., 2009). During capacitation, efflux of cholesterol induces aggregation of lipid raft microdomains into a large membrane raft (Aoki et al., 2005; van Gestel et al., 2005; Selvaraj et al., 2007). After capacitation, the uniform localization of lipid rafts in uncapacitated spermatozoa is replaced by a pattern of confinement with lipid rafts enriched with proteins known to take part in spermatozoa-ZP binding (Nixon and Aitken, 2009; Wang et al., 2020). There is also an increased presence of lipid rafts on the acrosomal region of the sperm plasma membrane (Nixon et al., 2009). These data indicate that the lipid rafts serve as a dynamic platform for relocation of proteins on the sperm plasma membrane during capacitation.

Our results demonstrated diminishment of C1orf56 immunoreactivities after acrosome reaction. The observation is highly suggestive that C1orf56 is present mainly on the plasma membrane, which is lost after acrosome reaction.

## Conclusion

Standard semen analysis provides limited information on sperm fertilizing capacity. Defective spermatozoa-ZP interaction can still occur in 13% men with normal semen analysis (Chiu et al., 2014). Until now, there is no simple method to identify spermatozoa with defective spermatozoa-ZP interaction. The present study tested the possibility of using C1orf56 expression on spermatozoa in predicting fertilization in clinical IVF. No difference in the C1orf56 expression on capacitated spermatozoa between the high FR group and the low FR group was found. The lack of difference between the two groups is likely due to the presence of multiple ZP receptors (Wassarman, 1999; Gadella et al., 2008; Fraser, 2010; Chiu et al., 2014) and reduction in one of them can be compensated by others. On the other hand, the C1orf56 level in the acrosome reacted spermatozoa was positively associated with fertilization rates. The observation suggests that C1orf56 may have functions after ZP-binding and acrosome reaction. However, the sample size in this study is small and a follow-up study with larger sample size is needed to confirm the observation.

Although assisted reproduction with intracytoplasmic sperm injection can improve fertilization, the cost and the associated risks of the micromanipulation procedure may not justify men with mild fertilization problems to undergo such treatment (Bhattacharya et al., 2001). To improve the clinical management of these men, it is important to diagnose defective ZP interaction with a reliable test before the commencement of assisted reproduction treatment. The determination of sperm ZP receptor can be a simple test for prediction of the fertilization potential of sperm samples in the future.

## Data Availability Statement

The mass spectrometry data is available at HKU Data Repository: https://doi.org/10.25442/hku.14677797.v1.

## Ethics Statement

The studies involving human participants were reviewed and approved by Ethics Committee of the University of Hong Kong/Hospital Authority Hong Kong West Cluster. The patients/participants provided their written informed consent to participate in this study.

## Author Contributions

YW, WZ, and SM: acquisition of data, analysis of data, and drafting the manuscript. PC and T-YL: analysis of data and critically revising the article. C-LL: analysis of data, critically revising the article, and teaching the technique. WY, J-PO, XL, and PC: study design, critically revising the article, and final approval of the version to be published. All authors have read and agreed to the published version of the manuscript.

## Conflict of Interest

The authors declare that the research was conducted in the absence of any commercial or financial relationships that could be construed as a potential conflict of interest.

## Footnotes

1 http://www.matrixscience.com

2 http://imagej.nih.gov/ij/index.html

3 https://doi.org/10.25442/hku.14677797.v1

4 http://www.proteinatlas.org/ENSG00000143443/normal
