# Supplementary material for: Identification of Sialyl-Lewis(x)-Interacting Protein on Human Spermatozoa
Source: Front Cell Dev Biol. 2021 Jul 20;9:700396. doi: 10.3389/fcell.2021.700396 (PMC8329450; doi:10.3389/fcell.2021.700396)
Supplement: Supplementary file 1 [file Table_1.DOCX]

**Supplementary Figure S1. Effects of different treatments on the acrosomal status of human spermatozoa. (A)** Capacitated spermatozoa were prepared by a 3-hour incubation in EBSS supplemented with 3% BSA. The percentage of capacitated spermatozoa was assayed by the chlortetracycline staining (CTC) method (N=10). Five CTC staining patterns of the sperm head were identified. Uncapacitated patterns were as follows: CTC1, a fluorescent band in the postacrosomal region; CTC2, a bright fluorescent head with a nonfluorescent postacrosomal region; and CTC3, a bright fluorescent head with a nonfluorescent thin band in the postacrosomal region. The capacitated pattern was CTC4, uniform head fluorescence. The acrosome-reacted pattern was CTC5, a decrease in or loss of uniform fluorescence over the head. Representataive photos of CTC staining are shown. **(B)** The percentage of acrosome reacted spermatozoa in the uncapacitated, capacitated and A23187-treated sperm population was detected by FITC-PSA staining (N=5). Data represent the mean ± SEM.

**
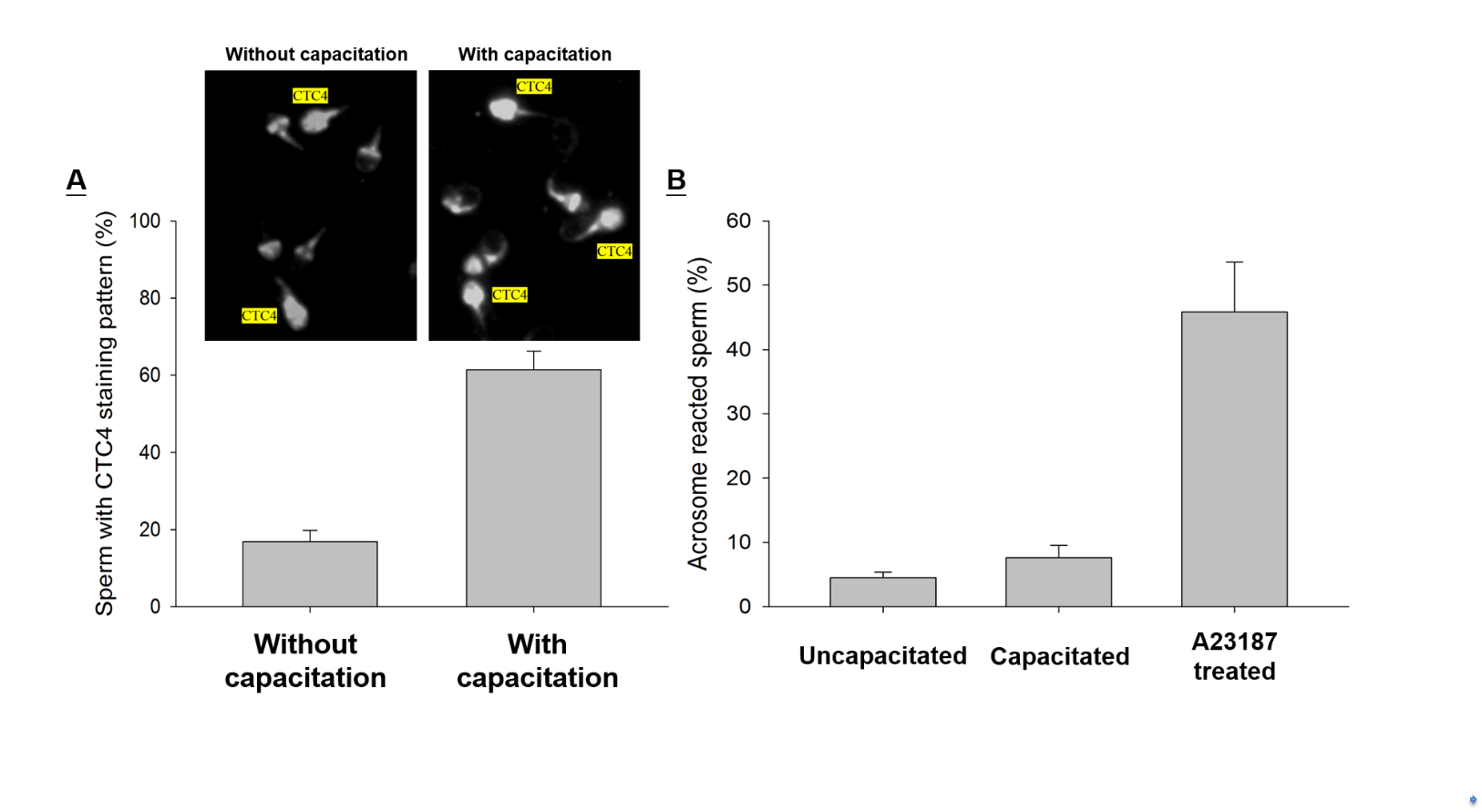
**

**Supplementary Figure S2. Simultaneous staining with antibody against C1orf56 and FITC-PSA.** The spermatozoa were mildly fixed, incubated with anti-C1orf56 and Alexa Fluor 555-conjugated goat anti-rabbit IgG, washed and further incubated with 1 FITC-PSA. The acrosome-reacted sperm without FITC-PSA staining were shown (arrows). Results shown are representative of 3 replicate experiments.


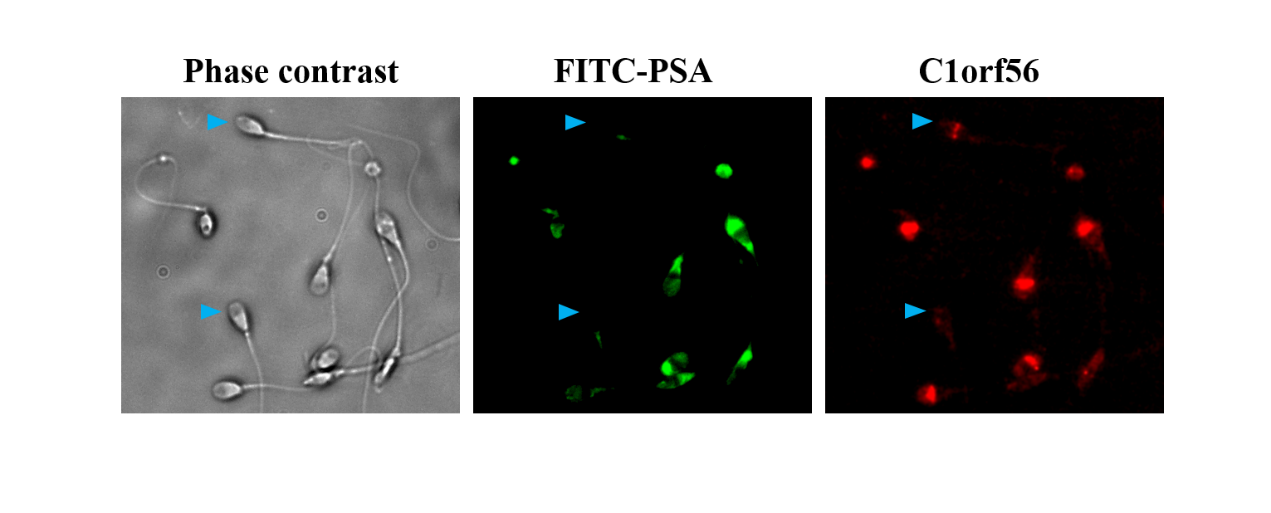


**Supplementary Figure S3. Effects of different concentrations of anti-C1orf56 blocking antibody on the sperm viability.** Sperm viability was determined by trypan blue exclusion test. Data represent the mean ± SEM (N=5).

| **Groups** | **Reagents used** | **Concentration**  **(μg/mL)** | **Viability** |
| --- | --- | --- | --- |
| 1 | - | - | 91.7±1.8 |
| 2 | Anti-C1orf56 antibody | 1 | 92.1±1.5 |
|  |  | 0.1 | 91.7±1.3 |
|  |  | 0.01 | 91.0±0.4 |
| 3 | Rabbit IgG | 1 | 91.7±1.1 |
|  |  | 0.1 | 90.8±1.6 |
|  |  | 0.01 | 92.3±0.7 |

**Supplementary Figure S4. Effects of different concentrations of C1orf56 blocking antibody on sperm motility.** Sperm motility parameters were determined by CASA system. Parameters including average path velocity (VAP), curvilinear velocity (VCL), straight line velocity (VSL), beat cross frequency (BCF), amplitude of lateral head displacement (ALH), linearity (LIN; VSL/VCL), straightness (STR; VSL/VAP) and progressive (PR) motility were measured. Data represent the mean ± SEM. *P < 0.05 when compared to the blank control (N=5).

| **Groups** | **Reagents used** | **Concentration**  **(μg/mL)** | **Sperm motility parameters (CASA)** | | | | | | | |
| --- | --- | --- | --- | --- | --- | --- | --- | --- | --- | --- |
|  |  |  | **VAP**  **(μm/s)** | **VCL**  **(μm/s)** | **VSL**  **(μm/s)** | **BCF**  **(Hz)** | **ALH**  **(μm)** | **LIN**  **(%)** | **STR**  **(%)** | **PR motility**  **(%)** |
| 1 | - | - | 59.24±5.5 | 106.1±14.5 | 48.78±5.5 | 6.32±0.8 | 30.74±3.1 | 86.2±2.6 | 48.8±5.2 | 36.8±8.4 |
| 2 | Anti-C1orf56 antibody | 1 | 48.42±7.6* | 83.54±14.2 | 42.36±6.4 | 4.82±0.6* | 30.92±1.8 | 88.8±1.5 | 57.6±3.9* | 47.6±3.6 |
|  |  | 0.1 | 50.58±8.2 | 97.08±1.9 | 43.66±6.9 | 5.16±1.0 | 30.96±2.1 | 87.8±2.5 | 55.2±4.9 | 41.4±14.4 |
|  |  | 0.01 | 55.96±10.5 | 104.76±8.1 | 50.5±10.9 | 5.54±0.6 | 30.2±0.6 | 86.4±1.6 | 52.2±5.1 | 40.4±12.8 |
| 3 | Rabbit IgG | 1 | 49.18±6.6* | 96.12±13.9 | 40.96±4.9 | 5.32±0.5 | 32.18±1.2 | 87.6±3.1 | 54.4±5.7 | 45.4±12.2 |
|  |  | 0.1 | 50.56±6.3 | 100.26±5.0 | 46.8±2.9 | 5.86±0.3 | 32.08±2.1 | 88.8±3.9 | 51.4±2.2 | 46.4±14.3 |
|  |  | 0.01 | 54.56±10.0 | 104.06±17.5 | 51.8±8.9 | 5.92±0.3 | 31.0±2.7 | 87.4±3.4 | 56.0±4.0 | 34.2±17.0 |

**Supplementary Figure S5. Effects of different concentrations of C1orf56 blocking antibody on the spontaneous and A23187-induced acrosome reaction of human spermatozoa.** Sperm acrosome status was detected by FITC-PSA staining and calculated the percentage of acrosome reacted spermatozoa. Data represent the mean ± SEM (N=5).

| **Groups** | **Reagents used** | **Concentration**  **(μg/mL)** | **Acrosome reacted spermatozoa (%)** | |
| --- | --- | --- | --- | --- |
|  |  |  | **Spontaneous**  **acrosome reacted** | **A23187-induced**  **acrosome reacted** |
| 1 | - | - | 16.8±3.1 | 76.8±4.4 |
| 2 | Anti-C1orf56 antibody | 1 | 16.1±1.5 | 77.4±1.8 |
|  |  | 0.1 | 14.7±0.7 | 74.7±1.2 |
|  |  | 0.01 | 14.9±3.0 | 72.7±2.8 |
| 3 | Rabbit IgG | 1 | 16.0±1.5 | 77.4±1.8 |
|  |  | 0.1 | 15.2±3.1 | 80.4±1.7 |
|  |  | 0.01 | 16.2±0.7 | 77.0±1.2 |

**Supplementary Figure S6. The relationship between C1orf56 expression and sperm motility.** C1orf56 surface expression is determined on (A) capacitated spermatozoa and (B) acrosome reacted spermatozoa by flow cytometry. Data was classified and analyzed according to the total motility, which is defined high motility group as the total motility>40% and low motility group as the total motility≤40%.

**A B**

**Supplementary Figure S7. The relationship between C1orf56 expression and morphology.** C1orf56 surface expression is determined on (A) capacitated spermatozoa and (B) acrosome reacted spermatozoa by flow cytometry. Data was classified and analyzed according to the sperm morphology, which is divided into normal morphology group and the abnormal morphology group. The samples were considered normal when the percentage of spermatozoa with normal morphology as defined by strict criteria is >4%

**A B**
